# *In situ* scarless skin healing of a severe human burn wound induced by a hCTLA4Ig gene-transferred porcine skin graft

**DOI:** 10.7150/ijms.62438

**Published:** 2022-05-21

**Authors:** Lijun Zhang, Guangchao Xu, Yating Wei, Mingzhou Yuan, Yuanyuan Li, Meifang Yin, Chufen Chen, Guangtao Huang, Bin Shu, Jun Wu

**Affiliations:** 1 Department of Burns and Plastic Surgery, the First Affiliated Hospital of Shenzhen University, Shenzhen Second People's Hospital, Shenzhen, P.R. China.; 2 Department of Burns, the First Affiliated Hospital, Sun Yat-sen University, Guangzhou, P.R. China.; 3 Human Histology & Embryology Section, Department of Surgery, Dentistry, Pediatrics & Gynecology, University of Verona Medical School, Italy.

## Abstract

Preventing fibrosis or hypertrophic scar formation following tissue damage is still a big challenge despite the numerous approaches clinicians currently use. Hitherto, no written account was available of a successful case of scarless skin healing after a severe burn injury. Here, we report the first case of the “perfect regenerative healing” of a severe burn wound with no hypertrophic scar formation in which a postage stamp skin autograft was covered with human cytotoxic-T-lymphocyte associated antigen4-immunoglobulin (hCTLA4Ig) gene-transferred pig skin. We also discuss the mechanisms involved in the scarless healing of human burn wounds.

## Introduction

Deep burns and traumata can cause devastating skin tissue damage often resulting in a life-threatening condition. In severe cases, wound coverage following excision and/or amputation can be problematic due to the limited availability of skin donor sites. In the clinical settings, allografting and xenografting are the current standard main approaches to temporarily cover extended wounds and thus prepare them for further treatments 1, 2.

It is well known that allografts and xenografts may provoke a strong immune response 3-6. While the rejection response happens within a short time, the dermal collagen of allografts and xenografts can survive for a somewhat longer period *in vivo* 7. Decellularized allografts and xenografts are commercially available which mitigate the otherwise strong immune response thus allowing to spread the use of allo-/xenografts. The latter have been applied for decades as covers and dermal substitutes in the clinical settings 6, 8-12. Decellularized xenografts are much more popular than allografts because of their unlimited supply from healthy animal donors. Skin grafts taken from pigs whose endogenous retrovirus (PERV) was inactivated via CRISPR-Cas9 13 can be the likely most favorable xenograft sources because the latter are similar to human skin in histological structure 6 and carry no risk of PERV transmission, while the pigs can be bred in large numbers 13, 14. On the other hand, with the development of biomedical engineering, cellular auto-/allografts have come into use, including cultured epidermal grafts 15, 16, keratinocyte allografts 17, fibroblast grafts 18, 19, ReCell systems 20, and human cytotoxic-T-lymphocyte associated antigen4-immunoglobulin (hCTLA4Ig) gene-transferred porcine skin 21-23. The function of these allo-/xenografts is to control the loss of vital fluids and proteins, mitigate pain, decrease the risk of infection, enhance tissue granulation, and epithelial cells growth 2. These devices supply the growth factors, cytokines, molecules, microstructures, and moist environments needed for cell and tissue regeneration in the course of wound healing. As additions to burn wound treatment, these material devices have found applications in leg ulcers 24, 25, keloids 26, complicated wounds 24, 27, bites 28, purpura fulminans 29, carcinoma, unstable scars 27, and bone defects 30. However, all the just mentioned skin replacements used to treat extended skin wounds have so far failed to regenerate either anatomically normal skin structures or cutaneous appendages, such as glands, nerves, and hair follicles. Hence, they have a limited capability of inhibiting the development of hideous scars 27. If either artificial or allo-/xeno-skin grafts could induce a tissue repair according to a natural scaffold prior to its degradation or immune rejection, the newly regenerated tissues might structurally and functionally “clone” the normal skin tissue. However, scarring could happen even in the autografts implanted group at the connecting edges between grafts and grafts and between grafts and normal skin. Moreover, a plain hypertrophic scar could form if the used split-skin autograft is not thick enough. In addition, grafting the wounds with meshed skin autografts usually gives rise to a mesh-like scar 31.

Developing an outstanding substitute which evokes little immune response but has the potential to induce the *in-situ* tissue regeneration with no scarring is still an enticing yet challenging dream. Interestingly, a promising example based on experiments of bone-induced regeneration shows that it is possible to achieve it in practice. Zhang and his team implanted calcium phosphate (Ca-P) bio ceramic materials *in vivo* to *de novo* regenerate a structurally normal bone tissue. The main requirement was to set into motion the body's own ability to regenerate bone tissues within the body environment. The Ca-P implanted material degraded and turned into a part of the body with no foreign body reaction and no fibrogenesis. Eventually, the authors achieved a perfect *in situ* repair of the bone proper structure and mechanics 31-33.

Here we report a severe skin burn wound case that partially healed with no scar due to a different local treatment. Our Burn Unit serendipitously discovered it when the patient came back four years after the burn injury for a follow-up check. Here, we will first detail the medical history, laboratory test results, and therapeutic management, including the determination of the healed skin origin, and later discuss the likely involved mechanisms.

## Case presentation and methods

### The case information

**Figure 1:**
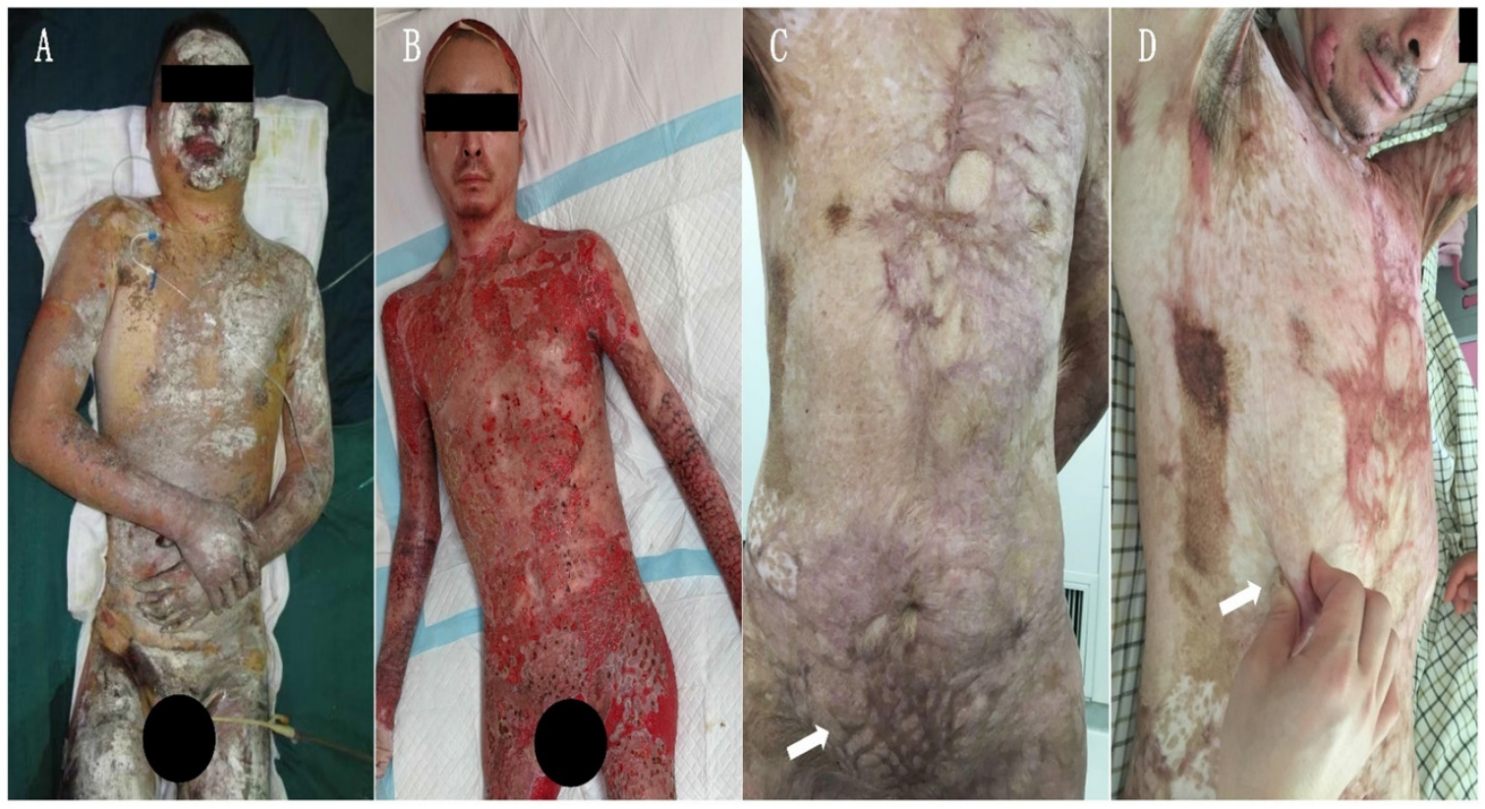
Images of the chest and abdomen burn wound of the patient.

**Figure 2:**
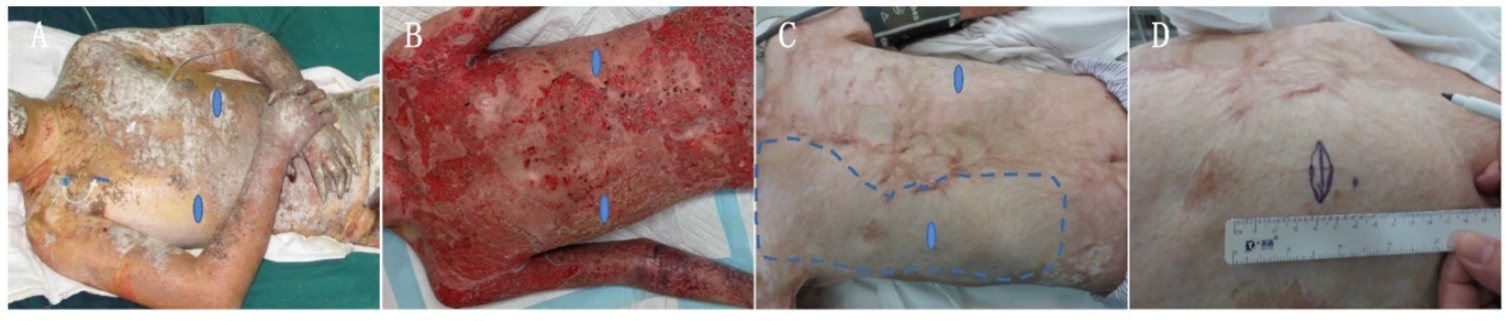
Images of the chest and abdomen wounds of the patient. The depth of the porcine skin covered area was deep-partial thickness and third-degree burns. The wound was debrided on day 30 post burns and the *hCTLA4Ig* gene-transferred porcine skin covered area was grafted on day 68 post burns. The blue dashed line shows the area initially covered with the *hCTLA4Ig* gene-transferred porcine skin. Blue oval-shape labelled areas show the sites where skin biopsies were collected.

In the year 2015, the Burn Center of the First Affiliated Hospital of Army Medical University (AMU) admitted a 34-year-old male patient burned by an electric arc flame. The wound area **(Figure 1A)** was 91% TBSA, including: 5% superficial II degree burn in parts of head, neck, and trunk; 28% deep II degree burn in parts of the head, neck, trunk, limbs, buttocks, and perineum; and 58% full thickness burn in hands, trunk, and limbs**.** The diagnosis included a moderate inhalation injury. After admission, the patient underwent a systematic medical treatment including fluid resuscitation, respiratory management, infection control, nutritional support, wound overseeing, and so on. As regards wound management, after 10 successive burn wound excisions, the application of autografts closed most of the deep thickness burn wounds. Due to the autografts limited availability, allografts harvested from the patient's daughter and son helped cover the upper front wounds of both thighs. In the last skin grafting operation, we found that postage stamp size skin autografts from the scalp previously applied to chest and abdomen were not completely confluent and a large area of granulation tissue between such grafts was exposed on the chest and abdomen right side **(Figure 2 B)**. Therefore, we applied two sheets of *hCTLA4Ig* gene-transferred porcine skin onto the chest and abdomen right side to cover the exposed wounds, protect them from infection, and promote their repair. The *hCTLA4Ig* gene-transferred porcine skin grafts attached to the wounds tightly and no rejection obtained during the entire process of wound repair. After 94 days of systematic treatment, the burn wound healed satisfactorily, and the patient went home. During the first year after his discharge he only discontinuously performed the prescribed rehabilitation exercises and anti-scar treatments, which included silicon hydrogel and pressure garments. Thereafter, he abandoned them because of personal reasons.

### Scarless wound healing (studied area) description

During a four-year follow-up, the patient reported a satisfactory wound repair and acceptable functional results at his right side of chest and abdomen. Obvious hypertrophic scars and a clear patchwork appearance developed at his right lower jaw, bilateral axillae, front and left chest sides, lower abdomen, and extremities **(Figure 1 C and D)**. The Vancouver Scar Scale (VSS) evaluation amounted to 6. Surprisingly, no hypertrophic scar developed at the right side of the chest and abdomen (studied area), that is at the sites first covered with postage stamp skin autografts and later with *hCTLA4Ig* gene-transferred porcine skin **(Figure 2 C. Blue dashed line)**. The regenerated skin in the studied area looked just like unburnt skin **(Figure 2 C and D).** There the patient lamented no pain and no itch. Under clinical assessment, the skin felt somehow rough, but its elasticity did not differ from that of normal skin **(Figure 1 D)**.

### Study design and methods

To throw light onto the mechanism(s) underlying the scarless healing at the patient's right side of chest and abdomen (studied area), we planned the following studies: (*i*) histological analysis to compare the structure of the studied area with those of the contralateral area, of the normal skin from an unburn volunteer, and of pig's skin; (*ii*) immunohistochemical staining to analyze the skin morphogenesis and differentiation of the studied area; (*iii*) whole genome sequencing (WGS) and mass spectroscopy (MS) to reveal the expression, if any, of porcine-originated genes and peptides in the studied area previously covered with the *hCTLA4Ig* gene-transferred porcine skin. Moreover, to investigate the function of the patient's immune system, the Hospital's Clinical Lab evaluated the (*iv*) immune cells ratio and proliferation, and (*v*) inflammatory cytokines. Finally, we also used the following experimental approaches: (*vii*) flow cytometry to classify the peripheral blood lymphocytes; (*viii*) phytohemagglutinin (PHA) and lipopolysaccharide (LPS) stimulation to evaluate the T cells and B cells function; and (*ix*) the mixed lymphocyte reaction (MLR) to test the response of mononuclear cell to cells of pig origin.

### Ethical approval

The Institutional Committee of Ethics (ICE) for Clinical Research and Animal Trials of the First Affiliated Hospital of Sun Yat-sen University approved all the procedures involved. The number of the Research Letter Approval is 2017-308. We made significant efforts to protect the patient's and volunteer's legitimate rights and interests by minimizing both the volume of blood samples and the size of skin samples used for the study. The patient and volunteer received clear and detailed information about all procedures, and both signed an informed consent.

### Preparation of porcine skin and blood samples

Our study used a 4-month-old female Bama mini pig acclimated for seven days prior to skin and blood samples collection. Anesthesia entailed an intramuscular injection of 30 mg/kg of a 1:1 combination of tiletamine and zolazepam. This donor pig was endotracheally intubated and kept under a surgical steady anesthesia with isoflurane 1.0-5.0% mixed with O_2._ Its back skin was prepped with Iodophor. A 2×3 cm (6 cm^2^) full thickness skin was excised and divided into two parts: the first was directly frozen in liquid nitrogen and next stored at -80 ℃ for RNA and DNA extraction; the second was fixed in 4% buffered formalin and next routinely embedded in paraffin. Five µm-thick sections underwent staining with hematoxylin and eosin (HE) and Masson trichrome. Blood (15 mL) from the carotid artery was taken and transferred to the Laboratory.

### Patient's biopsy and blood samples

Once admitted to the Department of Burns, the First Affiliated Hospital, Sun Yat-sen University, the patient underwent physical, electrocardiographic, and chest X-ray examinations before the skin and blood sample collection. The results of all such tests showed no pathological changes. Thereafter, surgery to take skin and blood samples was performed in the operation room under local anesthesia. The skin sample, 1×3 cm^2^, was taken from the right chest (**Figure 2**, blue oval-shape labelled areas, studied area, HP-R) just where a postage stamp skin autograft and sheets of *hCTLA4Ig* gene-transferred porcine skin had been sequentially applied. A control skin sample of the same size was collected from the left chest side (**Figure 2**, blue oval-shape labelled areas, control area, HP-L). The excision wounds were cosmetically sutured. The skin samples were cut into three parts: (*i*) two parts were immediately frozen in liquid nitrogen, next stored at -80 ℃, and later referred for WGS and MS to HaploX Genomics Center (Jiangxi, China) and Guangzhou Fitgene Biotechnology Company (Guangzhou, China); (*ii*) the third part was fixed in 4% buffered formalin and routinely embedded in paraffin; 5-µm-thick sections were stained with HE or Masson trichrome. The blood sample (30 mL) collected from a peripheral vein was divided in two parts: one for immune cells counting, cytokines testing, and immune function testing; and the other for routine blood testing.

### Immunohistochemical staining

Tissue samples were fixed using 4% paraformaldehyde and embedded in paraffin, and 5 μm sections of the paraffin‑embedded tissues were then used for immunohistochemical stainings, which was conducted using Super Plus TM Highly Sensitive and Rapid Immunohistochemical Kit (pH 9.0) (Cat. # E-IR-R220, Elabscience, China) according to the manufacturer's instructions. Briefly, the tissue sections were dewaxed, and antigens retrieved using the SP Reagent 9A (Dewaxing/Antigen Retrieval Buffer). Next, the endogenous peroxidase was quenched for 15 minutes at room temperature with SP Reagent B Peroxidase Blocking Buffer. The sections were then blocked for 30 minutes at 37 °C with normal goat serum (Boster, China), and subsequently incubated at 4 °C overnight with the primary antibody diluted with SP Reagent G Antibody Dilution Buffer at the respective optimized dilutions. The primary antibodies used were as follows: type Ⅰ collagen (COL1): Cell Signaling Cat. # 66948 (1:500); type Ⅲ collagen (COL3): Bioss bs-0549R (1:300); type Ⅳ collagen (COL4): Abcam Cat. # ab6586 (1:1000); type Ⅶ collagen (COL7): Boster Cat. # A011701-1 (1:1600); cytokeratin-10 (CK10): Abcam Cat. # ab76318 (1:20000); involucrin (IVL): Boster Cat. # BM1217 (1:800); and Ki 67: Cell Signaling Technology Cat. # 9449 (1:200). Finally, the sections were incubated for 30 min at room temperature with SP Reagent C Polyperoxidase-anti-Rabbit/Mouse IgG, followed by diaminobenzidine staining using the mixture of SP Reagent D and E. Then, SP Reagent F Hematoxylin Staining Buffer was used for counterstaining.

### Mass spectroscopy (MS)

The MS test was run as follows: (*i*) searching out the trusted collagen unique peptide in each test; (*ii*) searching out the common peptides shared by porcine tissue (P) sample and *hCTLA4Ig* gene-transferred porcine skin implanted sample (HP-R)( HP-R+P), and then subtracting the common peptides from HPL ((HP-R+P)-HP-L); next (*iii*) the peptides remaining after step (*ii*) were blasted to the human database to see whether there were duplicates with human peptides (((HP-R+P)-HP-L)-human database).

### Isolation of peripheral blood mononuclear cells (PBMCs), T cells and B cells

PBMCs from either humans or pig were isolated from whole‐blood samples via Ficoll (GE Healthcare) density gradient centrifugation. All cells were cultured in RPMI‐1640 Medium (Hyclone, USA), supplemented with 10% fetal bovine serum (FBS), antibiotics (streptomycin 100 U/mL and penicillin 100 U/mL) and 2 mM/L L-glutamine (Hyclone, USA) at 37 °C in a humidified atmosphere of 5% v/v CO_2_ and 95% v/v air. Following this, the isolation of T and B cells from PBMCs was done as previously described 1. Briefly, PBMCs underwent MACS^®^ microbeads (Miltenyi Biotec, Germany) isolation followed by use of Miltenyi Biotec kits to isolate T and B cells according to the producer's instructions.

### Proliferation assays of T cells and B cells

For proliferation assays we used Cell Counting Kit-8 (CCK8) from MCE (New Jersey, USA). We quantitatively evaluated the proliferation of T and B cells by following the product's instructions. Generally, the density of T and B cells was adjusted to 1×10^5^/mL. Next, 100 µL of T or B cells suspension were respectively seeded into 96-well plates and 100 µL of either 5 μg/mL PHA (Sigma) or 1μg/mL LPS (Sigma), respectively, was added to stimulate T cells and B cells growth. Then again, the CCK8 working solution (10μL) was added to each well followed by incubation of the plates for 4-h at 37 °C. Cells' harvesting occurred at days 1, 2, and 3. Finally, absorbances at 450 nm were measured using a microplate reader (Multiskan™ FC, Thermo, USA).

### Mixed lymphocytes reaction (MLR)

MLR was performed as detailed in an earlier study 32. Briefly, porcine PBMCs were treated with Mitomycin C (25μg/mL; MCE, New Jersey, USA) at 37 °C for 30 min. Next, they underwent three PBS washings to remove the Mitomycin. The number of treated PBMCs was adjusted to 1×10^6^/mL. Pre-treated porcine PBMCs (100µL) were cultured for 5 days together with an equal number of human PBMCs. Spent medium changes with fresh were done one every other day. Finally, cocultures were tested with CCK8 kits (MCE, New Jersey, USA) to assess human PBMCs proliferation.

## Results

### Histological analysis

**Figure 3:**
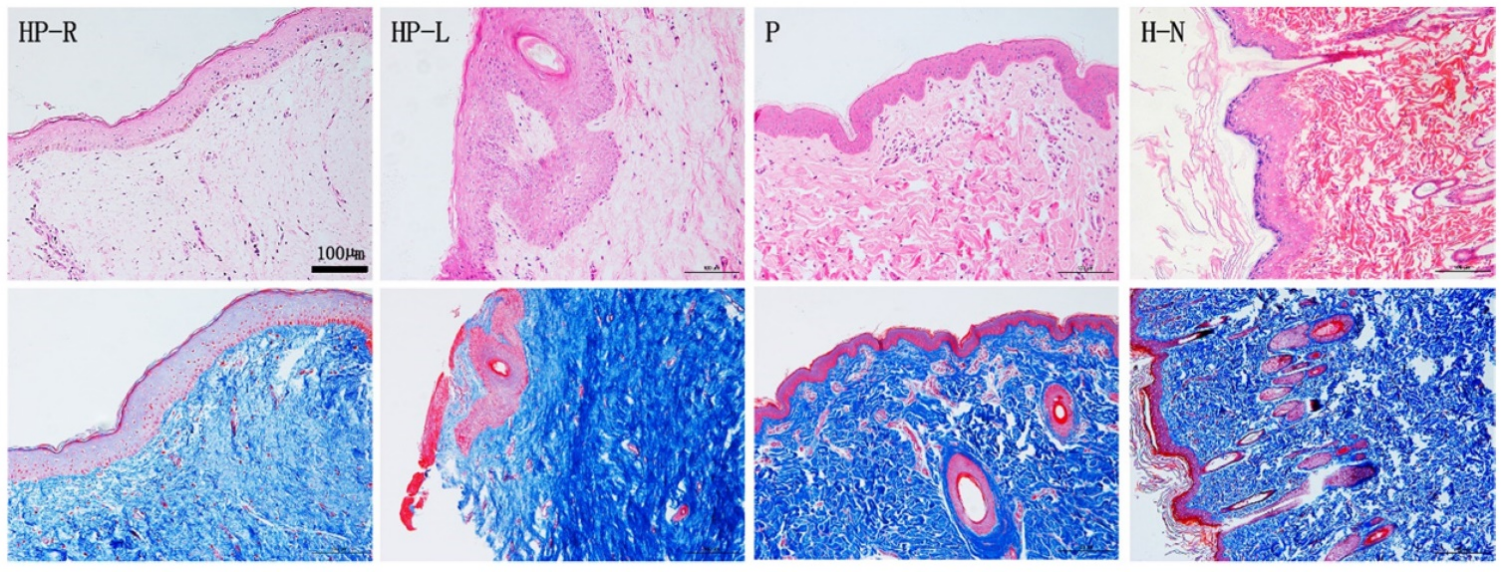
HE and Masson staining of skin tissue sections from the patient (HP-R and HP-L), a normal volunteer, and a donor pig. HP-R, the patient's right side chest skin tissue (studied area, scarless healed wound); HP-L, the patient's left side chest skin (control); P, pig (porcine) skin; H-N, human normal skin tissue from abdomen. Scale bar: 100 µm.

To detect the microscopic structure of the scarless healed wound (studied area; right side of chest and abdomen, HP-R), tissue sections underwent HE and Masson trichrome staining (**Figure 3**). Compared to the normal skin (H-N) from a volunteer and to samples of porcine skin (P), the studied area epidermis (HP-R) was smoother and exhibited less numerous papillary structures, and no skin appendages (**Figure 3 HP-R**). Masson trichrome staining revealed that collagen fibers assembled in bundles running parallelly to the epidermis; the subcutaneous tissue was shallow and loose; and collagen deposition was less abundant in the studied skin sample (HP-R) as compared to the patient's left side chest skin (HP-L).

### Immunohistochemical (IHC) staining

IHC stainings were performed to demonstrate any differences in the epidermis and dermis structure between the HP-R, HP-L, and normal skin. Type IV collagen (COL4) and type VII collagen (COL7) are identified as the principal structure of basement membranes 34. COL7 also forms anchoring plaques and anchoring fibrils, which importantly attach the epidermis to the dermis 35. IHC findings showed that COL7 was expressed in the basement membranes of all three skin samples. However, COL4 was almost negative. Ki67, CK10, and IVL were separately recognized as the markers of proliferation, early differentiation, and late differentiation, respectively. They were expressed in HP-R, HP-L and normal skin tissue samples. Interestingly, IVL was detected in the outer layer of the normal skin epidermis but, it was expressed in the whole epidermis of the HP-R and HP-L samples. COL1 was observed in all the HP-R, HP-L and normal skin sections. Conversely, COL3 was rarely detectable in all kinds of samples.

### WGS comparison

To find out whether any residual porcine skin had survived *in situ*, WGS was performed on both samples from the *hCTLA4Ig* gene-transferred porcine skin implanted (HP-R) area and the no porcine skin implanted left site (HP-L). By comparing both porcine and human databases, 93.232% and 93.076% reads of the tested genomics from HP-R and HP-L, respectively, were recognized as belonging to the human species, whereas very few reads of porcine genomics obtained from HP-R and HP-L samples **(Table 1A)**. Finally, a comparison of common porcine reads from both HP-R and HP-L was again performed with the human database. In the HP-R groups, 93.1132% of the tested samples were from human genomics and no porcine genomics could be detected. In the HP-L group, the respective results were 92.6016% and 0.1104% **(Table 1B)**. Altogether, these results showed that there was no significant difference in genomics consistence between the porcine skin implanted site and the no porcine skin implanted site, which further demonstrated that no porcine elements were still surviving in the scarless healed *hCTLA4Ig* gene-transferred porcine skin implanted site.

### MS examination

Conclusive results showed that no single porcine peptide could be detected in the *hCTLA4Ig* gene-transferred porcine skin implanted samples **(Table 2)**.

### Immune cells ratios and cytokines secretion

Immune cells ratios and cytokines secretion from the patient's PBMCs were assessed by the Clinical Laboratory of the First Affiliated Hospital, Sun Yat-sen University. All detected cells ratios were within normal ranges except for the regulatory T cells (Treg) ratio. According to available references, the normal range of the Treg proportion is 1.6%-6.14%^36^, ^37^. The observed proportion of this patient was 11.6% (**Table 3**).

The cytokines examined were all within reference ranges (**Table 4**).

### Patient's PBMCs exhibit a decreased immune response to stimuli *in vitro*

To evaluate the immune response of patient's PBMCs to different stimuli, we assayed the proliferation of T cells, B cells, and MLR *in vitro*.

**Figure 5:**
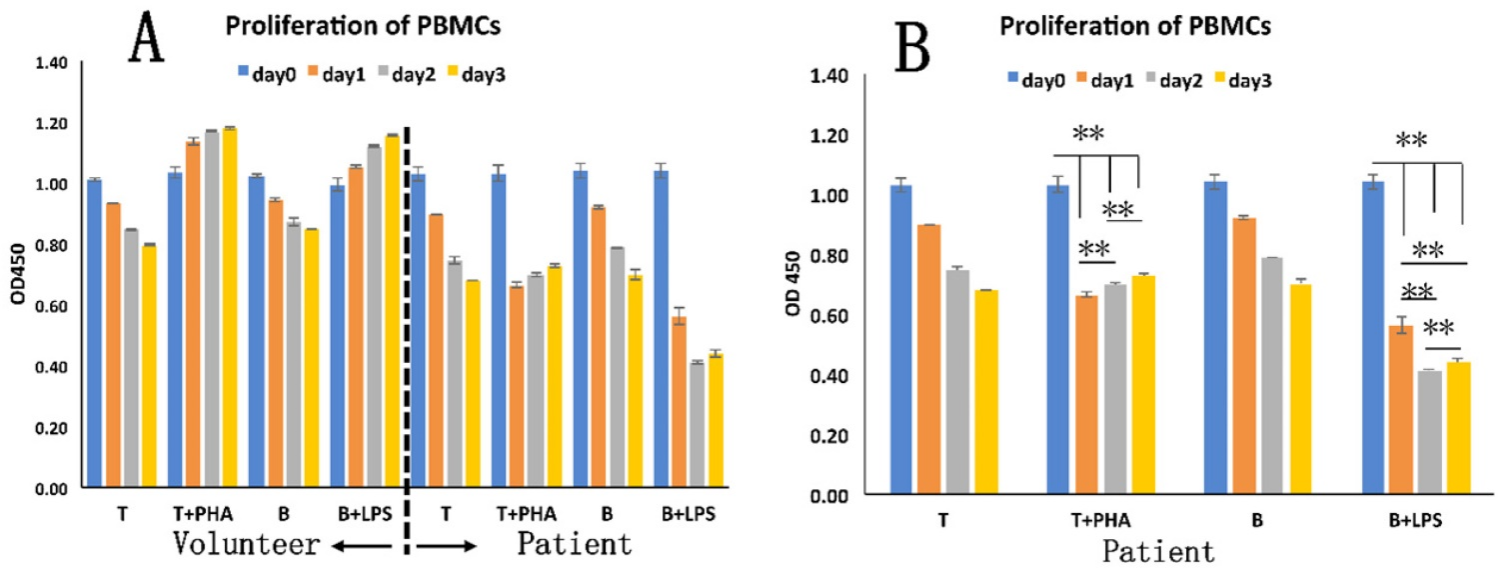
The immune response of patient's PBMCs. A: T and B cells from patient and healthy volunteer differently responded to PHA or LPS stimulation. B: Statistical analysis of different numbers of T and B cells from the patient. **, *p* < 0.01.

The patient's isolated T and B cells were separately pretreated with PHA or LPS **(Figure 5)**. A significantly decreased OD value (*p* < 0.01) was seen for the pre-treated cells (T cells, 1.03 ± 0.02; B cells, 1.04 ± 0.02) on day 1 (T cells, 0.89 ± 0; B cells, 0.56 ± 0.03) **(Figure 5B)**. At selected observation time points after stimulation, at day 2 or day 3, increased OD values occurred, which however were still lower than those of the pre-treated cells. The control PBMCs from the healthy exhibited the expected normal response to PHA **(Figure 5A)**.

**Figure 6:**
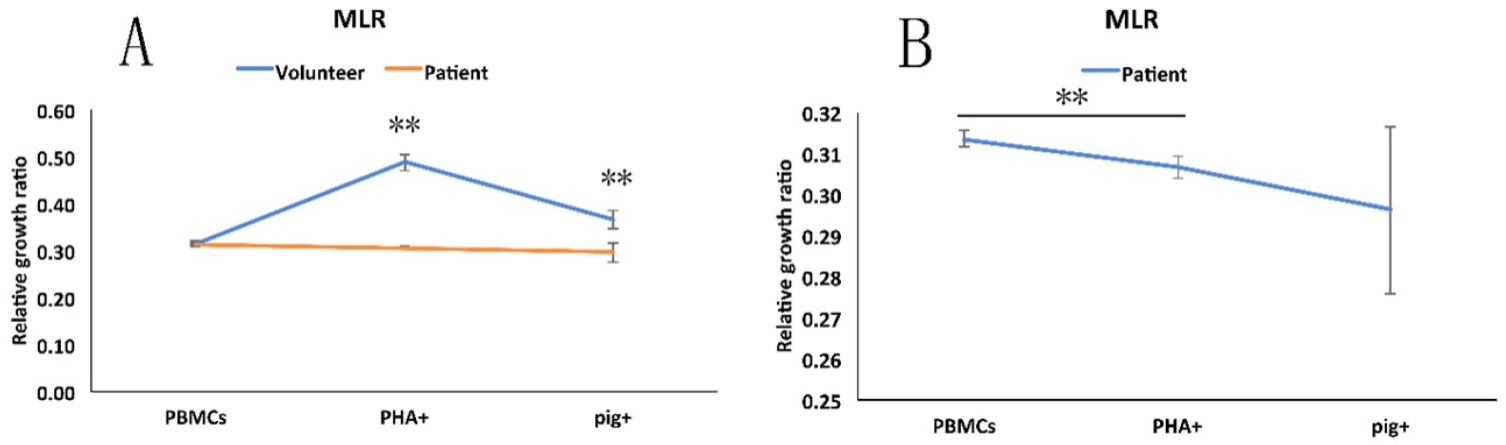
Mixed lymphocytes reaction (MLR). A: The different degrees of response of PBMCs from the patient, healthy volunteer to the pig's PBMCs and to added PHA. B: The statistical analysis of response of patient's PBMCs to PHA and pig's PBMCs. **, *p* < 0.01.

MLR showed that once stimulated by PHA *in vitro,* the patient's PBMCs proliferated less intensely than did the PBMCs from the healthy volunteer or from the pig **(Figure 6A).** Moreover, we found that PHA and the pig's PBMCs did not significantly stimulate the proliferation of the PBMCs from the patient **(Figure 6B)**.

## Discussion

To the best of our knowledge, this is the first report of a case of scarless and fully plastic healing of an extensive and deep burn wound. The involved patient's burn wound did heal after ninety-four days of hospitalization. Thereafter, we followed this patient up for two years by phone. In the year 2017, i.e. two years after the burn wound, the patient had a face-to-face interview with the follow-up doctor, who found a clear scar formation in the face, middle chest, left chest side, hypogastrium, and extremities **(Figures 1 and 2)**. To our surprise, the patient asked us to graft another pig skin to the left side of his chest and abdomen as we had done at the right side. Thus, we discovered the first “perfect (i.e. scarless) regeneration” instance of wound healing at the right side of his chest and abdomen, where no scar and no patchwork look had in the meantime developed. Moreover, the full skin plasticity had been conserved as well **(Figure 1D, *Arrow*)**. In comparison with the other scarred wound sites, after taking stock of a retrospective review of the patient's history, two things became clear, that is (*i*) the only different treatment at the right side of the chest and upper abdomen had been the inlaying of sheets of *hCTLA4Ig* gene-transfected porcine skin over a postage stamp skin autograft; and (*ii*) that the covering porcine skin sheets had not peeled off during the wound repair process. Furthermore, no acute and visible rejection phenomenon had occurred within the area of scarless wound healing. To investigate the mechanism of such a “perfect regeneration”, we spent another two years following the patient up by phone and performing the present study.

Previous investigations and clinical observations had shown that scar formation inevitably happens in deep second-degree and full-thickness burn wounds even when such wounds were treated with skin autograft and/or skin allo-/xenograft^31^. The scarring would come out from the edges of postage stamp or mashed skin grafts resulting in patchwork looks and meshed scars 38. On the other hand, no clinically successful immune tolerance induction to allo-/xenoskin without systemic immune suppression has been previously reported, as either alloskin or xenoskin grafts are eventually rejected 39. From the above facts, we raised the following questions regarding the occurrence of a scarless healing when a postage stamp skin autograft was put under the cover of *hCTLA4Ig* gene-transferred pig skin:

1. Is or is not the scarless healed wound still covered by *hCTLA4Ig* gene-transfected porcine skin, i.e. can an immune tolerance be induced towards this pig skin? Our results show that the answer is “no”. In fact, no porcine-derived specific proteins, peptides or gene fragments could be detected by whole genome sequencing (WGS) **(Table 1A-1B),** and mass spectroscopy (MS) **(Table 2)**. Then, what happened? The only possibility is that the recipient's own skin tissues replaced the pig's skin.

2. Is or is not the scarless healed area “normal”? The physical examination **(Figure 1D)** and histological results **(Figure 5)** prove that the regenerated dermal matrix looks structurally and functionally normal. However, the regenerated dermal tissue lacked rete pegs and cutaneous appendages. Interestingly, the same scarless healed wound area could function normally with neither itching nor pain.

3. How did the wound covered by *hCTLA4Ig* gene-transferred pig skin heal with no significant scar formation? Were the following factors involved in this phenomenon: (*i*) natural micro-templates; (*ii*) *in situ* living cells creating a proper micro-environment; and (*iii*) long-term surviving templates remaining *in situ* until regeneration was completed?

### A special immune environment prolonged the survival of porcine skin

In this case, the *hCTLA4Ig* gene-transferred porcine skin that covered the chest and abdomen right side did not peel off. Furthermore, no acute and visible rejection reaction occurred during the follow-up days. These phenomena show that the *hCTLA4Ig* gene-transferred porcine skin grafts could enjoy an extended survival time. But how could this porcine skin survive at the wound site without being rejected as usual? In addition to the well-known fact that *hCTLA4Ig* could inhibit T cell full activation 21, 40, here we evaluated the activity of the immune system by examining the functions of peripheral blood lymphocytes. Our results showed that the number of lymphocytes and amounts of cytokines they secreted were all within the normal range **(Tables 3 and 4)**. Interestingly, the proportion of Treg (11.7%) cells in this patient was two-fold higher than the normal range (1.6-6.14%) **(Table 3)**. Treg is recognized as the most important set of regulatory T lymphocytes as it can hinder an immune overresponse, inhibit an immune rejection, and block the production of some cytokines 41, 42. High Treg cells numbers can extend the survival time of transplanted organs 43. The much-heightened proportion of Treg cells in this patient might be the main cause of the long-term survival and of the invisible rejection of the covering porcine skin. Our MLR results also showed no obvious immune response of the patient's lymphocytes to PHA or pig lymphocyte stimulation **(Figure 6B)**, which suggested that the patient's immune system was “sluggish”. Last but not least, previous clinical studies showed that usually the immune system is to some extent dysfunctional in severely burnt patients (91% TBSA in this case) 6. Taken all the above findings together—particularly the higher Treg proportion and dysfunctional immune condition under the severely burnt condition—it is conceivable that the patient's immune characteristics and the local immune suppression contributed to the invisible rejection of the *hCTLA4Ig* porcine skin sheets grafted at the right chest and abdomen burn wound.

### An optimal degradation rate and a physiological template-based “dermal tissue structure-induced skin regeneration” led to the scarless wound healing

As we know, scar formation in deep burns is a common event because of the dermis damage. Even when wounds were covered with autograft skin, a significant scar would form at the junction of the implanted skin 39, just as happened in the hypogastrium of this patient **(Figure 1 C. Indicated by Arrow)**. An earlier study claimed that the autograft skin overlaid with porcine skin was an effective method to manage deep and large area burns. However, the researchers concluded that after scar maturation the patchwork appearance due to postage stamp grafts was acceptable. Nevertheless, the scar developed inevitably 38. Therefore, postage stamp skin autografts could hardly achieve a scarless healing in case of deep burn wounds.

How could a scarless healing have happened in this studied case? “Perfect regeneration” could be the exclusive explanation, i.e., newly formed skin tissues regenerated according to a perfect template *in situ* provided by the *hCTLA4Ig*-transferred porcine skin.

Transgenic porcine skin dermal matrix could have constituted a crawling trajectory for cell attachment, proliferation, differentiation, and could have also induced cells to infiltrate into the porcine skin graft 44**.** Previous studies showed that a dermal matrix can effectively reduce the severity of wound scars when used to cover deep second-degree burns 31. The involved mechanisms would entail that the dermal matrix could keep a stable internal environment by decreasing fluid losses and also exert the barrier action proper of a bio-membrane. Within the matrix, the amino acids of the collagen together with Zn^2+^ ions participate in tissue regeneration, promote surface epithelialization, and finally mitigate the scarring 45, 46. The extracellular matrix (ECM) of porcine skin could promote a vascular ingrowth from the wound's bed, induce a cellular infiltration from surrounding dermis, and keratinocytes' migration from the wound's edge. The utilization of ECM scaffolds could mitigate the wound contraction while not allowing the regeneration of the adnexa, such as sweat glands and hair follicles 47. Before being rejected by the host, the residual vascular networks within the porcine skin could help rebuild the blood circulation and hence the nutrients supply for cells and tissues in the wound. As a result of these positive factors, the longer-surviving porcine skin formed a suitable microenvironment which advanced the patient's skin cells and tissue regeneration and eventually resulted in a perfect wound repair with no typical scar formation. Simultaneously, the ECM was gradually and slowly wrapped by autologous cells and tissues 38. This could also explain why in this case no traces of the porcine skin could not be detected four years after implantation.

Therefore, the *hCTLA4Ig* gene-transfected porcine skin could have functioned as a physiological tissue regeneration template. This process can be named as “dermal tissue structure-induced skin regeneration”.

Actually, the concept of “induced regeneration” was first proposed in the bone regeneration field by Zhang and his team 48. In their studies, calcium phosphate (CaP) bioceramic materials enclosed by swaths of fresh autogenic periosteum carrying osteoblasts and other multipotent cells were implanted into bone defect sites which later on resulted occupied by bone tissue endowed with Haversian systems 49. In their following study, biphasic calcium phosphate (BCP) bioceramics composed of micro-whiskers and nanoparticles with a hybrid-structured surface (hBCP) were implanted *in vivo* and formed new bone tissue. In an *in vitro* study, the same authors found that hBCP induced a dramatic downregulation of the inflammatory response 50. CaP bioceramics were gradually replaced by bone tissue *in vivo*, eventually achieving bone regeneration 51. A number of studies reported that nanosized CaP materials exerted positive effects on cartilage regeneration 52-54, angiogenesis and vasculogenesis 55, 56, and nerve tissue regeneration 57 as well. Collectively, these results indicated that scaffolds with nanosized structure do positively influence cellular attachment and subsequent biological behavior, including adsorption of proteins, cell adhesion, cellular responses 58, 59, degradation of biological compounds, and osteoblasts' gene expression 60-63. According to all the above-mentioned findings, Zhang proposed the novel concept of “tissue-induced biomaterials”, which broke through the traditional notion that inanimate materials could not induce the regeneration of living tissues and organs.

In addition to hard tissue induced regeneration, a soft tissue could also induce holomorphosis. In a nerve regeneration study, Gu and his team proposed the idea of “constructing biodegradable tissue engineered nerves”, using chitosan materials combined with bone marrow mesenchymal stem cells to induce peripheral nerve regeneration and experimental recovery of limb function 63-67. Professor Gu's team believes that controlling the biodegradation rate of chitosan nerve conduits is extremely important for repairing nerve defects of varying length, location and diameter 66, 68, 69.

**Figure 7:**
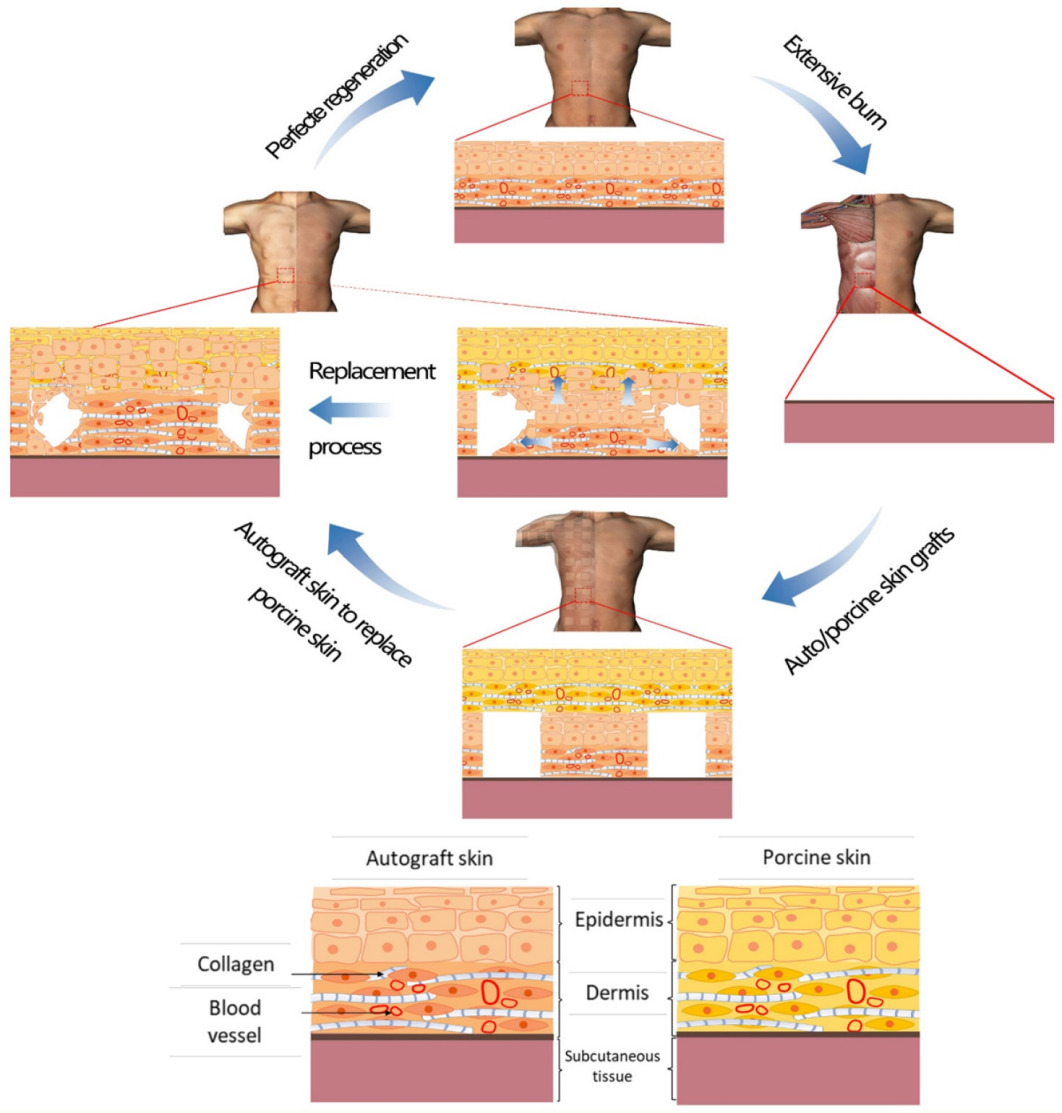
Schematic representation of the mechanism that could explain the “dermal tissue structure-induced skin regeneration”. After an extensive burn, an autoskin graft was implanted onto the wound and next a *hCTLA4Ig* gene transfected porcine skin sheet was inlaid onto it, which supplied suitable microenvironmental conditions and matrix spatial structure for an *in situ* scarless tissue regeneration. Finally, the wound closed with absolutely no scar formation and no residual porcine skin components.

In all the above-mentioned studies, the implanted materials were gradually replaced by the recipient's cells and tissues with no visible rejection. The main mechanism involved is that implanted materials stimulate an extremely limited immune response, while having enough time to act as scaffolds for tissue regeneration. Implanted materials also create a suitable microenvironment for cell attachment, proliferation, and differentiation, followed by the scaffold's gradual degradation and replacement by the host's cells and tissues. Similarly, the operative mechanism of scarless healing in this case was that of “dermal tissue structure-induced skin regeneration”. *hCTLA4Ig* gene-transfected porcine skin supplied suitable microenvironment conditions and matrix spatial structures for neoangiogenesis, cell attachment, proliferation, differentiation, and matrix remodeling during its prolonged covering of the wound. In the meantime, the porcine skin was gradually degraded and replaced by the patient's regenerating cells and tissues according to the template's physiological structure. Regenerated skin tissue thus could form a physiological structure with a normal appearance. The upshot was a scarless healed wound with no residual traces whatsoever of porcine skin. Therefore, we would posit that this occurrence is a typical case of “dermal tissue structure-induced skin regeneration” **(Figure 7)**.

## Conclusions

Perfect skin regeneration with no scar formation after a severe burn wound is possible via an *in situ* natural template-induced physiological-structural regeneration pathway. The long-term survival of a natural template (in this instance pig skin endowed with living cells) is the prerequisite. Further studies are needed to make this beneficial situation generally applicable in the proper clinical settings.

## Figures and Tables

**Figure 4:**
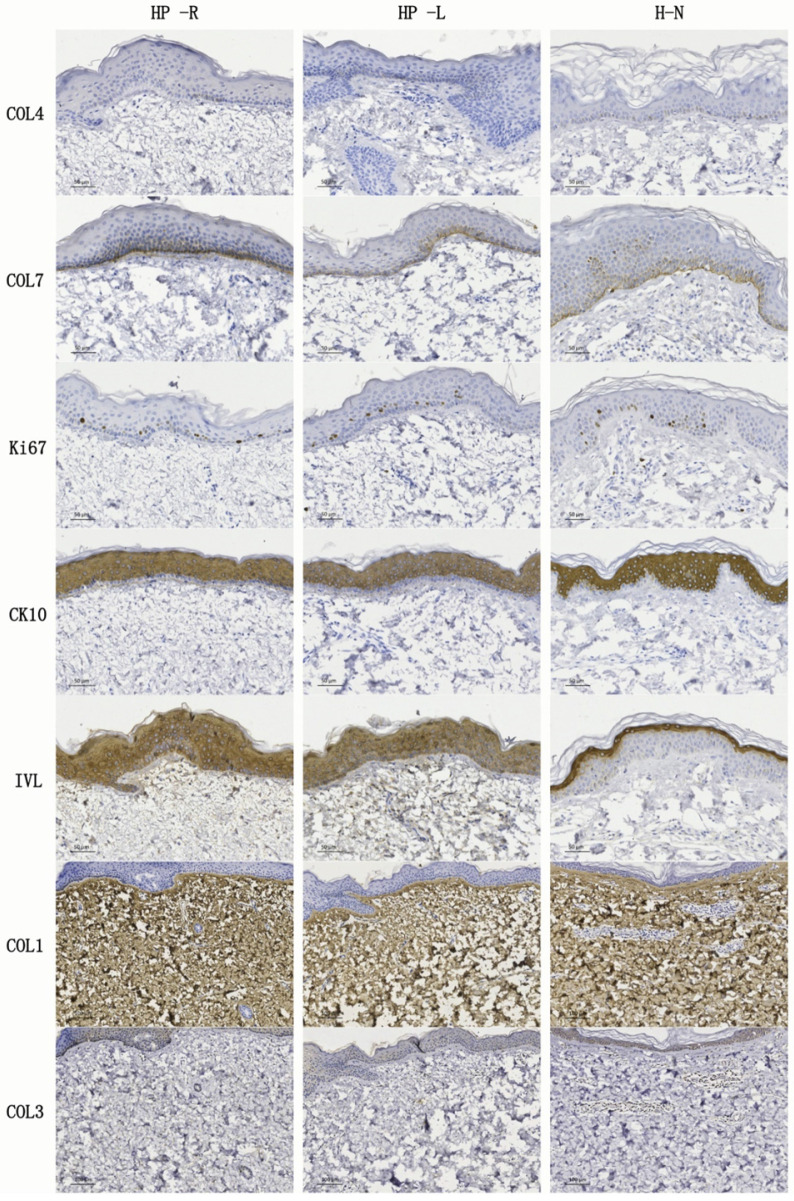
IHC stainings of skin tissue sections from the patient (HP-R and HP-L) and from a normal volunteer. COL4 and COL7 were detected in the basement membranes. Ki67 was expressed in the epidermis and the most positive cells were located near the basement membrane, while CK10 and IVL were expressed within the epidermis. COL1 was present in all the HP-R, HP-L and normal skin samples. Conversely, COL3 was rarely detectable in any of the samples. HP-R, the patient's right side chest skin tissue (studied area, scarless healed wound); HP-L, the patient's left side chest skin (control); H-N, human normal skin tissue from abdomen. Scale bars: 50 µm or 100 µm.

**Table 1A:** Take 100,000 reads from HP-R and HP-L as compared with porcine database and human database.

Samples	Reads	Species	Mapped Rate (%)
HP-L	93076	s__Homo sapiens	93.076
	2	s__Sus scrofa	0.002
HP-R	93232	s__Homo sapiens	93.232
	0	s__Sus scrofa	0

**Table 1B:** Take common porcine reads from HP-R and HP-L as again compared with the human database.

Samples	Reads	Species	Mapped Rate (%)
HP-L	8386	s__Homo sapiens	92.6016
	10	s__Synthetic construct	0.1104
HP-R	8518	s__Homo sapiens	93.1132
	7	s__Synthetic construct	0.0765

**Table 2:** The number of trusted unique collagen peptides in tested samples.

Samples	HP-R	HP-L	P	HP-R+P	(HP-R+P)-HP-L	((HP-R+P)-HP-L)-human database
Number of peptides	220	329	220	23	4	0

HP-R+P: common peptides in HP-R and P. (HP-R+P)-HP-L: peptides from the common peptides in HP-R and P not found in HP-L. ((HP-R+P)-HP-L)-human database: the remaining peptides form (HP-R+P)-HP-L were not found in the human database. HP-R, the patient's right-side chest skin tissue (studied area, scarless healed wound). HP-L, the patient's left side chest skin of the. P, porcine skin.

**Table 3:** Immune cell proportion in peripheral blood

Items		Results	unit	Reference ranges
T cells	CD3+	62.0	%	62.0-76.0
	CD3+ CD69+	2.0	%	
Th cells ^a^	CD3+ CD4+	33.5	%	32.0-46.0
	CD3+CD4+CD69+	0.1	%	
Ts cells ^b^	CD3+CD8+	24.8	%	18.0-32.0
	CD3+CD8+CD69+	0.6	%	
B cells	CD19+CD69	1.0	%	
	CD19+	13.6	%	7.0-18.0
NK cells ^c^	NK cell	20.1	%	7.0-18.0
	CD3-16+ or CD56+69+	0.6	%	
Treg ^d^		11.6	%	

^a,^ Th cells, T helper cells; ^b,^ Ts cells, suppressor T cells; ^c,^ NK cells: Natural killer cells; ^d,^ Treg: regulatory T cells.

**Table 4:** Quantifications of cytokines in the supernatant of cultured peripheral blood immune cells

Items	Results	Unit	Reference ranges
IL^a^-2	0.49	pg/mL	0.00-5.71
IL-4	0.80	pg/mL	0.00-2.80
IL-6	0.99	pg/mL	0.00-5.30
IL-10	0.89	pg/mL	0.00-4.91
TNF^b^	0.58	pg/mL	0.00-2.31
IFN-γ^c^	0.53	pg/mL	0.00-7.42

^a,^ Interleukin; ^b,^ Tumor necrosis factor; ^c,^ Interferon
